# Gastrointestinal manifestations and nutritional therapy during COVID-19 pandemic: a practical guide for pediatricians

**DOI:** 10.31744/einstein_journal/2020RW5774

**Published:** 2020-07-07

**Authors:** Jane Oba, Werther Brunow de Carvalho, Clovis Artur Silva, Artur Figueiredo Delgado

**Affiliations:** 1 Hospital Israelita Albert Einstein São PauloSP Brazil Hospital Israelita Albert Einstein, São Paulo, SP, Brazil.; 2 Hospital das Clínicas Faculdade de Medicina Universidade de São Paulo São PauloSP Brazil Instituto da Criança e do Adolescente, Hospital das Clínicas, Faculdade de Medicina, Universidade de São Paulo, São Paulo, SP, Brazil.

**Keywords:** Coronavirus infections/complications, COVID-19, Betacoronavirus, SARS-CoV-2, Gastrointestinal diseases/etiology, Nutritional therapy, Inflammatory bowel diseases, Child, Adolescent

## Abstract

Coronavirus disease 2019 (COVID-19) is a disease caused by the severe acute respiratory syndrome coronavirus 2 (SARS-CoV-2), which has spread globally in pandemic proportions. Accumulative evidence suggests SARS-CoV-2 can be transmitted through the digestive system, the so-called fecal-oral route of transmission, and may induce several gastrointestinal manifestations. MEDLINE^®^ and Embase databases were extensively searched for major clinical manifestations of gastrointestinal involvement in children and adolescents with COVID-19 reported in medical literature, and for nutritional therapy-related data. Findings and recommendations were pragmatically described to facilitate overall pediatric approach. A total of 196 studies addressing gastrointestinal or nutritional aspects associated with the global COVID-19 pandemic were found. Of these, only 17 focused specifically on pediatric patients with regard to aforementioned gastrointestinal or nutritional aspects. Most articles were descriptive and six addressed guidelines, established protocols, or expert opinions. Children and adolescents with gastrointestinal symptoms, such as nausea, vomiting and diarrhea, should be seriously suspected of COVID-19. Gastrointestinal signs and symptoms may occur in 3% to 79% of children, adolescents and adults with COVID-19, and are more common in severe cases. These include diarrhea (2% to 50%), anorexia (40% to 50%), vomiting (4% to 67%), nausea (1% to 30%), abdominal pain (2% to 6%) and gastrointestinal bleeding (4% to 14%). Patients with inflammatory bowel disease or chronic liver disease are not at greater risk of infection by SARS-CoV-2 relative to the general population. Nutritional support plays an important role in treatment of pediatric patients, particularly those with severe or critical forms of the disease. The digestive system may be a potential route of COVID-19 transmission. Further research is needed to determine whether the fecal-oral route may be involved in viral spread. Nutritional therapy is vital to prevent malnutrition and sarcopenia in severe cases.

## INTRODUCTION

Coronavirus disease 2019 (COVID-19) is caused by a single-stranded RNA severe acute respiratory syndrome coronavirus 2 (SARS-CoV-2). The disease was first described in Wuhan, China, on December 31, 2019, and quickly spread throughout the world. On March 11, 2020, the World Health Organization (WHO) declared COVID-19 a pandemic. As of May 11, 2020, the cumulative number of confirmed cases and deaths reported in five continents and 215 countries amounted to more than 4 million and 283**,**271, respectively.^(1)^ In Brazil, there have been 177**,**589 confirmed cases and 12,400 confirmed deaths, and figures continue to rise.^(2)^ However, confirmatory testing for the disease is limited among the Brazilian population.

The clinical spectrum of COVID-19 in adults, children and adolescents ranges from asymptomatic infection to severe pneumonia, acute respiratory failure, multiple organ dysfunction syndrome and fatal illness.^(3)^ Children and adolescents may exhibit milder symptoms compared to adults, and tend to respond favorably to treatment, achieving symptom resolution in a short period of time.^(4)^ Recently, digestive symptoms related to the COVID-19 have been emerged as an extrapulmonary symptom, concerning specialists, mainly pediatricians.^(5-7)^ SARS-CoV-2 RNA has also been detected in the feces of patients affected with COVID-19 pneumonia, suggesting the presence of live virus in feces and potential fecal-oral transmission.^(8)^ Growing clinical evidence suggest the digestive system may indeed be an alternative route of infection.

Digestive symptoms, such as abdominal pain and diarrhea, are particularly common in pediatric patients. Therefore, nutritional therapy plays a significant role in prevention of further undernutrition and sarcopenia in COVID-19 infected patients. Finally, children and adolescents with comorbidities or preexisting chronic diseases, such as inflammatory bowel (IBD) or liver disease, including those undergoing immunosuppressive or biological treatment, may be at higher risk of developing severe forms of COVID-19 with gastrointestinal manifestations.

The objective of the present study was to describe the main clinical gastrointestinal manifestations in children and adolescents with COVID-19, and to investigate the role of nutritional therapy in prevention of malnutrition and sarcopenia in these patients.

All aspects of gastrointestinal involvement in children will be emphasized in this literature review, as follows: viral transmission route, mechanisms of infection, general clinical features, gastrointestinal manifestations (including IBD- and liver disease-related symptoms), diagnosis and selected therapeutic approaches (with particular emphasis on nutritional therapy).

MEDLINE^®^ and Embase medical databases were extensively searched using the search terms “COVID**-**19”, “gastrointestinal disease”, “nutrition”, “nutritional therapy”, “immunosuppression”, “IBD”, “children” and “adolescents”. Articles addressing specific aspects of the pediatric population are scarce. Available articles are mostly descriptive and based on gastrointestinal manifestations associated with the recent global pandemic. Guidelines, treatment protocols and expert opinions, although rarely reported, were prioritized.

Findings and recommendations were pragmatically described to facilitate overall pediatric approach.

The studies were analyzed according to the following items: viral transmission route; mechanisms of SARS-CoV-2 infection; clinical features of COVID-19 infection; gastrointestinal manifestations (including IBD- and liver disease-related symptoms); nutritional involvement and recommendations; and laboratory data.

More than 11**,**509 studies associated with COVID-19 were found. Studies investigating gastrointestinal or nutritional aspects associated with the global COVID-19 pandemic (196) were analyzed. Only 17 studies were specific to the pediatric population regarding the above mentioned gastrointestinal or nutritional aspects. Most articles were descriptive, and six articles addressed guidelines, established protocols or specialist opinions.

The authors of this study provided a critical appraisal of publications and avoided simple reproduction of data.

## VIRAL TRANSMISSION ROUTE

In children and adolescents, SARS-Cov-2 transmission occurs mainly via respiratory droplets, aerosols and the conjunctiva, possibly the same transmission routes described in adults.^(4)^ Interhuman transmission occurs via droplets and direct or indirect contact with surfaces. Children and adolescents are a vulnerable group due to close contact with family members. The virus has been isolated from serum, blood, rectal swabs, saliva, urine and feces.^(9)^ SARS-CoV-2 RNA has also been detected in the feces of adult and pediatric patients, 1 to 12 days after testing negative in respiratory samples. Another interesting finding is that stool samples collected from children remain positive for longer periods of time compared to adults.^(10)^ This is a strong indication of potential oral-fecal viral transmission. However, mechanisms underlying gastrointestinal symptoms remain elusive.

According to Cruz et al., symptomatic and particularly asymptomatic children may be vectors and shed the virus in feces in the community, at school or at home.^(11)^ Asymptomatic children may be a source of prolonged transmission to adults, and further research is warranted to determine the viability and infectivity of SARS-CoV-2 in feces, so as to control the spread of the virus.

Perinatal aspects of COVID-19 are poorly understood. To date, there is no evidence of intrauterine vertical transmission to infants born to mothers infected with SARS-CoV-2 during pregnancy,^(12)^ but this possibility cannot be ruled out. Most neonates at risk of COVID-19 development investigated to date had mild respiratory distress, low incidence of feeding intolerance and favorable outcomes.^(13)^ Also, SARS-CoV-2 does not seen to be excreted in breast milk. In a study with breast milk samples collected from six mothers, specimens tested negative for SARS-CoV-2. However, small sample size in those studies preclude conclusive statements.^(9,14)^

## MECHANISM OF SARS-COV-2 INFECTION IN CHILDREN

Many infectious diseases have different manifestations in children and adolescents relative to adults. The reason why COVID-19 is less severe in children remains to be determined. It is also uncertain whether the inflamed gut, as in colitis, provides an optimal doorway through which the virus may enter human cells, or if immunosuppressive therapy is associated with increased risk of SARS-CoV-2 infection in children and adolescents.^(15)^ Related data are scarce.

The key to human transmission is the ability of coronavirus to bind to cells. SARS-CoV-2 infects primarily respiratory epithelial cells and spreads to other organs. The angiotensin converting enzyme receptor 2 (ACE2) is vital for COVID-19 infected cells and is highly expressed in lung cells and enterocytes in the ileum and colon. The virus uses ACE2 to invade cells. Zhu et al., reported higher ACE2 activity in children and adolescents aged 4 to 13 years and progressive decrease to adult levels after this age.^(15,16)^ They postulated that children and adolescents may be less susceptible to SARS-CoV-2 infection due to differences in ACE2 activity and immunity. It has also been suggested that SARS-CoV-2 may spread via the fecal route, since ACE2 is expressed in lung epithelial cells and enterocytes in the ileum and colon.^(5,17,18)^

Cytokine storm syndrome is thought to be a major pathogenetic mechanism in multiple organ failure associated with SARS-CoV-2 infection.^(18)^ Indeed, high levels of expression of cytokines and chemokines, such as interleukin-6 (IL-6), have been reported in COVID-19 patients and were found to be positively correlated with disease severity. However, target cells, pathogenetic mechanisms and transmission routes have not as yet been fully elucidated and many points remain to be clarified.

## CLINICAL FEATURES OF COVID-19

COVID-19 affects primarily the adult population and causes a series of respiratory conditions. The most common symptoms associated with initial SARS-CoV-2 infection are fever, cough, fatigue, myalgia and shortness of breath, with 10% to 20% of patients progressing to severe acute respiratory syndrome (SARS).^(19,20)^ Risk factors for poor outcomes include advanced age (over 60 years) and presence of comorbidities (heart disease, hypertension, history of stroke, *diabetes mellitus* and chronic lung or kidney disease).^(14,21)^ Signs and symptoms of COVID-19 in children and adolescents range from asymptomatic to acute respiratory failure and are less frequent and severe compared to adults.^(4)^ Fever, cough, shortness of breath, myalgia and fatigue are the most common symptoms described in pediatric populations, with severe disease accounting for 2% of cases.^(4,14)^ Interestingly, according to the Centers for Disease Control and Prevention (CDC), symptoms are common to Chinese and American children.^(21)^ Risk factors for severe illness in children include chronic lung disease, cardiac, neuromuscular or genetic diseases and immunosuppression.^(22)^ However, infants appear to be more vulnerable to SARS-CoV-2 infection and have higher rates of serious illness compared to older children. Indeed, the reported prevalence of severe or critical disease in children and adolescents aged <1 year, 1 to 5 years, 6 to 10 years, 11 to 15 years or 16 to 17 years upon diagnosis corresponds to 10.6%, 7.3%, 4.2%, 4.1% and 3.0%, respectively.^(4)^

Hyperinflammatory shock with features akin to atypical Kawasaki disease (also known as Kawasaki disease shock syndrome and toxic shock syndrome) has been described in some COVID-19 cases in England and the United States. Most cases shared similar clinical presentation, with unrelenting fever (38°C -40°C), variable rash, conjunctivitis, peripheral edema, generalized distal limb pain and significant gastrointestinal symptoms. Progression to refractory shock and cardiac compromise is common in unusual specific respiratory conditions.^(23,24)^

## GASTROINTESTINAL MANIFESTATIONS OF COVID-19 INFECTION

Gastrointestinal signs and symptoms may affect 3% to 79% of children, adolescents and adults with COVID-19, and are more common in severe cases.^(6,24)^ Manifestations include diarrhea (2% to 50%), anorexia (40% to 50%), vomiting (4% to 67%), nausea (1% to 30%), abdominal pain (2% to 6%) and gastrointestinal bleeding (4% to 14%). Vomiting is more often reported in pediatric populations, whereas diarrhea is the most common symptom in both children and adults.^(25)^ In the absence of respiratory symptoms, diarrhea may be the first symptom prior to diagnosis in 22% of cases. Yellow, watery diarrhea with stool frequency ranging from three to nine per day and average duration of 4 days has been described. Laboratory fecal tests usually reveal low leukocyte count and no blood, supporting viral infection.^(25)^

Diarrhea caused by SARS-CoV-2 does not seems to damage the colonic epithelium. Likewise, lymphocytic inflammatory infiltration may eventually be found in the esophagus, stomach, colon and liver of adult patients with COVID-19.^(5)^ This finding suggests gastrointestinal symptoms reflect immune response rather than organ damage. Assuming SARS-CoV-2 may in fact spread via the fecal route, Xiao et al., emphasized the significance of stool specimen screening with reverse transcription polymerase chain reaction quantitative real time (rRT-PCR) in prevention of fecal-oral transmission.^(5)^ Pediatricians and gastroenterologists must also remain vigilant about variant conditions mimicking gastroenteritis, other viruses (*e.g*., adenovirus), viral hepatitis, dengue and gastrointestinal adverse events associated with immunosuppressive therapy and use of biologic agents.

## INFLAMMATORY BOWEL DISEASE AND LIVER DISEASE

Inflammatory bowel disease includes Crohn’s disease and ulcerative colitis. Children with IBD often present with more extensive and severe disease than adults. Therefore, IBD specialists have been challenged to address specific aspects of SARS-CoV-2 infection in pediatric IBD patients, particularly those undergoing immunosuppressive or biological therapy. Data on pediatric IBD patients infected with SARS-CoV-2 are scarce. National and international guidelines and consensus suggest patients with IBD are not at greater risk of infection with SARS-CoV-2 relative to the general population. In children and adolescents with IBD and under immunosuppressive therapy who have digestive symptoms and COVID-19, it is reasonable to investigate other causes and exclude aforementioned enteric infections first. Gastrointestinal pathogens such *Clostridioides difficile* should then be excluded. Finally, active inflammation should be confirmed based on calprotectin and C-reactive protein levels, or cross-sectional imaging findings. Inflammatory bowel disease should be managed according to disease activity and severity and treatment includes immunosuppressive and biological therapies. Corticosteroids may be used to treat episodes of clinical relapse, provided they are given in low doses for short periods of time and tapered as soon as possible.^(26,27)^ Provisional guidance from the Porto group of ESPGHAN suggests that, in children and adolescents with IBD, the disease *per se* is not a risk factor for SARS-CoV-2 infection or more severe disease development relative to the general population.^(27)^ In order to prevent IBD adverse outcomes, intestinal inflammation must be controlled and therapies maintained.

Whether children and adolescents with IBD or liver transplanted who are receiving immunosuppressive and biological treatment are more susceptible to SARS-CoV-2 infection remains to be determined and so far no specific recommendations have been made by any of the two societies.^(26,27)^ No severe disease manifestations (acute respiratory failure and/or multiple organ dysfunction syndrome) or deaths have been reported among immunosuppressed pediatric IBD and liver transplant patients so far. These findings reflect the initial experience and are therefore subject to change as data accumulates. General and specific recommendations and therapeutic guidelines for pediatric IBD patients are listed in tables 1 and 2, respectively.^(26-29)^

**Table 1:** Recommendations for children and adolescents with inflammatory bowel disease during the COVID-19 pandemic

**General**

Wash hands frequently
Maintain social distancing (minimum distance of 1m)
Cover mouth and nose when coughing or sneezing
Wear face masks
If you have fever, cough or difficulty breathing, seek medical care as soon as possible
Face-to-face visits should be replaced with telemedicine whenever possible
Medical staff should monitor patients with active disease or flare via telephone calls
Physical exercise is essential and may be practiced at home via the internet
Psychological/ psychiatric support may be required

**Specific**

At this stage, IBD does not seem to be a risk factor for SARS-CoV-2 infection in children and adolescents
Adherence to drug therapy should be reinforced
Vaccination should be updated, particularly annual influenza vaccination
Active IBD disease should be treated according to standard IBD care, since risks outweigh the risk of COVID-19-related complications

**Infusion centers during the COVID-19 pandemic**

In stable children, switching from infliximab to adalimumab should be discouraged unless intravenous infusions cannot be provided
Combination biologic/immunomodulatory therapy may carry a higher risk compared to monotherapy
Elective endoscopic examinations and surgical procedures should be postponed during the epidemic
Colonoscopy should be replaced with fecal calprotectin test.

Source: adapted from Turner D, Huang Y, Martín-de-Carpi J, Aloi M, Focht G, Kang B, Zhou Y, Sanchez C, Kappelman MD, Uhlig HH, Pujol-Muncunill G, Ledder O, Lionetti P, Dias JA, Ruemmele FM, Russell RK; Paediatric IBD Porto group of ESPGHAN. Corona Virus Disease 2019 and Paediatric Inflammatory Bowel Diseases: Global Experience and Provisional Guidance (March 2020) from the Paediatric IBD Porto Group of European Society of Paediatric Gastroenterology, Hepatology, and Nutrition. J Pediatr Gastroenterol Nutr. 2020;70(6):727-33;^(27)^ Queiroz NS, Barros LL, Azevedo MF, Oba J, Sobrado CW, Carlos AS, et al. Management of inflammatory bowel disease patients in the COVID-19 pandemic era: a Brazilian tertiary referral center guidance. Clinics (Sao Paulo). 2020;75:e1909.^(29)^

IBD: inflammatory bowel disease; SARS-CoV-2: severe acute respiratory syndrome coronavirus 2.

**Table 2:** Therapy-specific considerations for pediatric inflammatory bowel disease patients during the COVID-19 pandemic

**Aminosalicylate acid derivatives (sulfasalazine)**

No evidence of increased risk of COVID-19 infection
Should never be discontinued

**Corticosteroids**

Safety during COVID-19 infection is unclear
Systemic corticosteroids are not thought to provide clinical benefits
Corticosteroids may be used to treat relapse episodes in low doses and for short periods of time. Taper as soon as possible

**Immunomodulators (thiopurines and methotrexate)**

No evidence of increased risk of COVID-19 infection
Immunomodulators have been prescribed in standard doses or intervals to almost all children
SARS-CoV-2 Positive and Negative (symptomatic): discontinuation of immunosuppressive therapy is recommended during acute febrile illness and should only be reintroduced when fever subsides, and the child regains normal health^(27)^
Resume treatment two weeks after sign and symptom resolution
SARS-CoV-2 Positive (asymptomatic): Therapeutic decisions should be made on an individual basis^(26)^

**Anti-TNF therapy**

Only Infliximab and Adalimumab have been approved
No evidence of increased risk of COVID-19 infection
Maintain dose and infusion intervals
SARS-CoV-2 positive and asymptomatic: Biological therapies should be delayed for 2 weeks to monitor for COVID-19 symptoms and resumed after signs and symptoms have subsided^(26)^
Switching from infliximab to adalimumab should be discouraged in stable patients

Source: adapted from Rubin DT, Feuerstein JD, Wang AY, Cohen RD. AGA clinical practice update on management of inflammatory bowel disease during the COVID-19 Pandemic: expert commentary. Gastroenterology. 2020 Apr 10:S0016-5085(20)30482-0. doi: 10.1053/j.gastro.2020.04.012. [Epub ahead of print];^(26)^ Turner D, Huang Y, Martín-de-Carpi J, Aloi M, Focht G, Kang B, Zhou Y, Sanchez C, Kappelman MD, Uhlig HH, Pujol-Muncunill G, Ledder O, Lionetti P, Dias JA, Ruemmele FM, Russell RK; Paediatric IBD Porto group of ESPGHAN. Corona Virus Disease 2019 and Paediatric Inflammatory Bowel Diseases: Global Experience and Provisional Guidance (March 2020) from the Paediatric IBD Porto Group of European Society of Paediatric Gastroenterology, Hepatology, and Nutrition. J Pediatr Gastroenterol Nutr. 2020;70(6):727-33;^(27)^ Queiroz NS, Barros LL, Azevedo MF, Oba J, Sobrado CW, Carlos AS, et al. Management of inflammatory bowel disease patients in the COVID-19 pandemic era: a Brazilian tertiary referral center guidance. Clinics (São Paulo). 2020;75:e1909.^(29)^

SARS-CoV-2: severe acute respiratory syndrome coronavirus 2; TNF: tumor necrosis factor.

## NUTRITIONAL INVOLVEMENT AND RECOMMENDATIONS

Interestingly, in children and adolescents with severe forms of COVID-19, acute onset pro-inflammatory cytokine storm may lead to undernutrition and sarcopenia within a short period of time. Sarcopenia has been associated with postoperative mortality or morbidity in several surgical procedures.^(30,31)^

Diarrhea and vomiting have been described in 9% and 7% of patients, respectively, and are thought to be play an important role in undernutrition development, a risk factor in critically ill patients.^(30)^ Still, as previously alluded to, the significance of potential fecal transmission remains unclear, as well as the relationship between ACE2 expression in the digestive system and viral invasion of epithelial cells.^(14,32,33)^

Weight loss due to lack of appetite is common in adult patients (78.5%) and may aggravate nutritional status in severe forms of disease. Anosmia may impair taste perception and interfere with nutritional supplement or food intake.^(33,34)^

COVID-19 associated shock or severe clinical condition should not be seen as a contraindication to trophic enteral nutrition. In children and adolescents with significant respiratory involvement who require ventilatory support, excessive swallowing of air and further gastric distention may occur.^(15,30)^ This condition may predispose to gastroesophageal reflux and aspiration pneumonia associated with mechanical ventilation. Pulmonary infection may induce delayed gastric emptying and intestinal hypomotility, leading to constipation. Such clinical conditions may preclude immediate delivery of nutritional therapy. Also, measurement of feeding tolerance based on residual gastric volume or other indicators is highly controversial and whether this feeding intolerance is impacted by patient type (surgical or nonsurgical) remains to be determined. Association of other clinical parameters (abdominal distention, vomiting and normal bowel sounds) is more reliable. However, gastric feeding is preferred to optimize digestive activity and the post-pyloric route (preferably jejunal) is recommended in case of failure. Prokinetic agents are recommended to enhance motility prior to this intervention. Health professionals should handle aerosol generating procedures carefully (post-pyloric tubes carry a higher risk). Polymeric diets (lactose-free or not) and special oligomeric nutritional therapy (primarily via post-pyloric administration) are commonly used. Associations between continuous enteral nutrition and diarrhea reduction have been emphasized in some studies.^(15,30,35-37)^

Lipid-enriched diets may satisfy caloric requirements and prevent essential fatty acid depletion and excessive carbon dioxide production. Fiber-free formulas may be better tolerated in the presence of significant gastrointestinal dysfunction. There is no evidence that omega 3, arginine or glutamine supplementation may translate into better nutritional progression or prognosis in pediatric populations.^(15,30,38,39)^

In patients with COVID-19 and severe respiratory compromise, overfeeding should be avoided due to higher risk of hypercapnia. Prone positioning is not a contraindication for early enteral nutrition, which has been associated with a better prognosis.^(14,31)^

Minimum protein levels ranging from 1 to 2.5g/kg per day must be provided to prevent protein hypercatabolism.^(14)^ Patients with COVID-19 may have higher micronutrient requirements. However, further supplementation with high doses of vitamins (including A, C, D and E) or other oligo elements, such as zinc and selenium, is not recommended. Studies investigating micronutrient requirements in adults with COVID-19 are controversial.^(15,39)^

Parenteral nutrition therapy should be avoided except in moderately to severely undernourished patients with intestinal failure. Gastric administration facilitates earlier initiation of feeding. Early enteral nutrition seems to be the best recommendation to ensure adequate anthropometric progression in pediatric COVID-19 patients. Sufficient food supply can only be determined in sequential nutritional evaluations including clinical and laboratory findings.^(15,39)^ As previously alluded to, breast milk is a true immunonutrient and its administration must be encouraged.

To date, nutritional therapy recommendations are mostly based on observations, rather than evidence derived from clinical trials, and should follow basic critical care nutrition principles.^(14)^

During the SARS-CoV-2 pandemic, nutritional needs of vulnerable children and adolescents must be met to prevent exacerbation of disparities in health and educational achievements. Longer screen time, irregular sleep schedule, poorer diets leading to weight gain, and loss of cardiorespiratory fitness are secondary effects of school closure and home confinement in children.^(36,40,41)^

## COVID-19 RELATED LABORATORY DATA

Some specific laboratory findings are worthy of notice in COVID-19 patients. Elevated aminotransferase levels are observed in 20% to 30% of patients. Hypoproteinemia and prolonged prothrombin time have also been reported during COVID-19 infection. Lower monocyte count and higher prevalence of antimicrobial treatment have been reported in adult COVID-19 patients with digestive symptoms, relative to those with no digestive symptoms. Lymphocytopenia, a poor prognostic factor, seems to be less common in children.^(30,37,38)^

Elevated C-reactive protein, procalcitonin, cytokine, ferritin, triglyceride and D-dimer levels have been described in aforementioned cases of hyperinflammatory shock, with laboratory evidence of infection or severe inflammation.^(24)^

## DISCUSSION AND CRITICAL APPRAISAL

Digestive symptoms did not receive much attention at the beginning of the COVID-19 pandemic. As the pandemic progressed and cumulative data were collected on a global scale, it became evident that digestive symptoms are indeed very common.^(5,6)^

Detection of SARS-CoV-2 in stool samples and other clinical specimens led to increased awareness of the risk of disease transmission via the fecal-oral route. It has also been suggested that the digestive system may be a potential route for COVID-19 infection transmission, particularly among children and adolescents, although this hypothesis remains to be confirmed. Also, stool samples collected from children remain positive for the virus for longer periods of time relative to adults.^(7,8,39)^

Children and adolescents with chronic gastrointestinal diseases, such as IBD and liver disease, may be at higher risk of COVID-19 infection development compared to healthy individuals of similar age. However, international surveillance databases, guidelines and provisional consensus suggest these diseases are not a risk factors in these patients.^(27,28)^ Cumulative data are needed for further clarification.

Children and adolescents with COVID-19, particularly those with severe forms of the disease, may quickly progress to nutritional deterioration. Early delivery of enteral nutrition via carefully placed gastric tube to minimize personal contamination risks is paramount. Parenteral nutrition compounds the risk of prolonged length of hospital stay due to secondary infection. Sarcopenia with poor prognosis may develop in response to longer mechanical ventilation time. Nutritional therapy may improve prognosis and recovery, and there is no evidence of higher risks of persistent diarrhea or gastrointestinal manifestations, provided adequate diets are provided.^(40-42)^

The digestive system may be a potential route for COVID-19 transmission. Children and adolescents with gastrointestinal symptoms, such as vomiting and diarrhea, should be seriously suspected of having COVID-19. Further research is needed to determine whether fecal-oral transmission could be an additional viral transmission route. Still, potential fecal shedding by infected patients should be considered.

Chronic diseases, such as inflammatory bowel and liver disease, do not seem to be a risk factor for COVID-19. Likewise, discontinuation of immunosuppressive therapy in affected patients does not seem appropriate now.

Nutritional therapy is vital for prevention of malnutrition and sarcopenia in severe cases. Further studies investigating gastrointestinal manifestations, laboratory findings and imaging abnormalities in large populations of children and adolescents with COVID-19 are warranted.
