# Supplementary material for: Gastrointestinal manifestations and nutritional therapy during COVID-19 pandemic: a practical guide for pediatricians
Source: Einstein (Sao Paulo). 2020 Jul 7;18:eRW5774. doi: 10.31744/einstein_journal/2020RW5774 (PMC7346091; doi:10.31744/einstein_journal/2020RW5774)
Supplement: Translation

# Translation: Sintomas gastrintestinais e abordagem nutricional durante a pandemia de COVID-19: guia prático para pediatras

**Authors:** Jane Oba, Werther Brunow de Carvalho, Clovis Artur Silva, Artur Figueiredo Delgado

**Affiliations:** 1 Brasil Hospital Israelita Albert Einstein, São Paulo, SP, Brasil.; 2 Brasil Instituto da Criança e do Adolescente, Hospital das Clínicas, Faculdade de Medicina, Universidade de São Paulo, São Paulo, SP, Brasil.

**Keywords:** Infecções por coronavírus/complicações, COVID-19, Betacoronavírus, SARS-CoV-2, Gastroenteropatias/etiologia, Terapia nutricional, Doenças inflamatórias intestinais, Criança, Adolescente

## Abstract

A doença pelo coronavírus 2019 (COVID-19) é causada pelo coronavírus da síndrome respiratória aguda grave 2 (SARS-CoV-2) e foi amplamente disseminada em todo o mundo em proporções pandêmicas. Evidências crescentes sugerem que o sistema digestivo pode ser uma via potencial para a infecção pelo SARS-CoV-2, para a disseminação do vírus por via fecal-oral, e estar relacionado com vários sintomas gastrintestinais. Realizamos uma extensa revisão da literatura médica utilizando os bancos de dados MEDLINE^®^ e Embase, com o objetivo de identificar as principais manifestações clínicas do envolvimento gastrintestinal e analisar a terapia nutricional em crianças e adolescentes com COVID-19. Os achados e as recomendações foram descritos de maneira pragmática, para facilitar a abordagem do pediatra em geral. Foram analisados 196 estudos relacionados ao envolvimento do trato gastrintestinal ou aspectos nutricionais associados à pandemia de COVID-19 em todo o mundo. Destes estudos, apenas 17 incluíram a população pediátrica exclusivamente com aspectos gastrintestinais ou nutricionais específicos. Os artigos, em sua maioria, foram descritivos, sendo seis relacionados a diretrizes, protocolos instituídos ou opiniões de especialistas. Crianças e adolescentes com sintomas gastrintestinais, como náusea, vômito e diarreia, devem ser avaliados como pacientes suspeitos de COVID-19. Os sinais e sintomas gastrintestinais podem ocorrer em 3% a 79% das crianças, adolescentes e adultos com COVID-19, estando mais frequentemente presentes em casos graves. Incluem diarreia (2% a 50%), anorexia (40% a 50%), vômitos (4% a 67%), náusea (1% a 30%), dor abdominal (2% a 6%) e sangramento gastrintestinal (4% a 14%). Pacientes com doença inflamatória intestinal ou doenças hepáticas crônicas não apresentam maior risco de infecção por SARS-CoV-2 do que a população em geral. O suporte nutricional é parte muito importante do tratamento de pacientes pediátricos, principalmente nas formas graves ou críticas da doença. O trato gastrintestinal pode ser uma via potencial para a infecção por COVID-19. Mais pesquisas são necessárias para determinar a possibilidade da transmissão fecal-oral, importante para a disseminação viral. A terapia nutricional é essencial para prevenir desnutrição e sarcopenia nos casos graves.

## INTRODUÇÃO

A doença causada pelo novo coronavírus (COVID-19) decorre da infecção pelo coronavírus da síndrome respiratória aguda grave 2 (SARS-CoV-2). A doença foi descrita pela primeira vez em 31 de dezembro de 2019, em Wuhan, na China, e se disseminou mundialmente de forma rápida. Em 11 de março de 2020, a Organização Mundial da Saúde (OMS) declarou a pandemia de COVID-19. Em 11 de maio de 2020, o número acumulado de casos e óbitos confirmados notificados em cinco continentes e 215 países ultrapassou 4 milhões e 283.271, respectivamente.^(1)^ No Brasil, houve 177.589 casos e 12.400 óbitos confirmados, e os números seguem aumentando.^(2)^ Entretanto, a testagem confirmatória da doença é limitada na população brasileira.

O espectro clínico da COVID-19 em adultos, crianças e adolescentes varia de infecção assintomática a pneumonia grave, insuficiência respiratória aguda, síndrome da falência múltipla de órgãos e doença fatal.^(3)^ Em crianças e adolescentes, os sintomas podem ser comparativamente mais brandos, e a resposta ao tratamento tende a ser favorável, com resolução dos sintomas em período curto de tempo.^(4)^ Sintomas extrapulmonares da COVID-19 envolvendo o sistema digestivo foram recentemente relatados e são motivo de preocupação entre especialistas, principalmente entre pediatras.^(5-7)^ Recentemente, o RNA do SARS-CoV-2 foi detectado nas fezes de pacientes com pneumonia decorrente de COVID-19, sugerindo a presença de vírus vivo nas fezes e a possibilidade de transmissão fecal-oral.^(8)^ Evidências clínicas crescentes sugerem que o sistema digestivo seja, de fato, uma via alternativa de infecção.

Dor abdominal e diarreia são sintomas digestivos bastante comuns em pacientes pediátricos. Assim, a terapia nutricional tem papel importante na prevenção da desnutrição e da sarcopenia em pacientes infectados. Finalmente, crianças e adolescentes com comorbidades ou doenças crônicas preexistentes, como a doença inflamatória intestinal (DII) ou doença hepática, assim como os que estão sob tratamento imunossupressor ou biológico, podem ter maior risco de desenvolvimento de formas graves de COVID-19 com manifestações gastrintestinais.

Este estudo teve por objetivo descrever as principais manifestações clínicas de envolvimento gastrintestinal em crianças e adolescentes com COVID-19 e investigar o papel da terapia nutricional na prevenção da desnutrição e da sarcopenia.

Todos os aspectos do envolvimento gastrintestinal em crianças são enfatizados nesta revisão narrativa, incluindo vias de transmissão viral, mecanismos de infecção, características clínicas gerais, manifestações gastrintestinais (inclusive sintomas relacionados à DII e à doença hepática), diagnóstico e abordagens terapêuticas selecionadas (com ênfase na terapia nutricional).

As bases de dados médicas MEDLINE^®^ e Embase foram objetivo de ampla busca, empregando-se os unitermos “COVID-19”, “doença gastrointestinal”, “nutrição”, “terapia nutricional”, “imunossupressão”, “DII”, “crianças” e “adolescentes”. Artigos abordando aspectos específicos da população pediátrica são escassos, e os existentes são principalmente de caráter descritivo e baseados em manifestações gastrintestinais associadas à recente pandemia global. As diretrizes, os protocolos de tratamento e as opiniões de especialistas, embora raramente relatados, foram priorizados.

Os achados e as recomendações foram descritos de forma pragmática, para facilitar a abordagem do pediatra em geral.

Os estudos foram analisados de acordo com os itens: via de transmissão viral; mecanismos de infecção por SARS-CoV-2; características clínicas da infecção por COVID-19; manifestações gastrintestinais (incluindo as relacionadas à DII e à doença hepática); envolvimento nutricional e recomendações e dados laboratoriais.

Foram encontrados mais de 11.509 estudos relacionados à COVID-19. Aqueles que investigaram o envolvimento gastrintestinal ou aspectos nutricionais associados à pandemia global de COVID-19 (196) foram analisados. Somente 17 estudos abordaram especificamente a população pediátrica com relação aos aspectos gastrintestinais ou nutricionais descritos. A maioria dos artigos era de caráter descritivo e seis abordavam diretrizes, protocolos estabelecidos ou opiniões de especialistas.

Os autores deste estudo fizeram uma avaliação crítica das publicações, evitando a simples reprodução do conhecimento.

## VIA DE TRANSMISSÃO VIRAL

O SARS-CoV-2 é transmitido em crianças e adolescentes principalmente por meio de gotículas respiratórias, de aerossóis e da conjuntiva – possivelmente as mesmas vias descritas em adultos.^(4)^ A transmissão inter-humana se dá por meio de gotículas e contato direto ou indireto com superfícies. Crianças e adolescentes constituem um grupo vulnerável, devido ao contato próximo com membros da família. O vírus foi isolado a partir do soro, sangue, swabs retais, saliva, urina e fezes.^(9)^ O RNA do SARS-CoV-2 também foi detectado nas fezes de adultos e crianças, 1 a 12 dias após terem testado negativo em amostras respiratórias. Outro dado interessante é que amostras de fezes coletadas de crianças permanecem positivas por mais tempo do que as de adultos.^(10)^ Esse achado é um forte indicativo de transmissão oral-fecal. Entretanto, os mecanismos envolvidos no desenvolvimento de sintomas gastrintestinais permanecem desconhecidos.

Segundo Cruz et al., crianças sintomáticas e, sobretudo, as assintomáticas, podem ser vetores e excretar o vírus nas fezes na comunidade, na escola ou em casa.^(11)^ Crianças assintomáticas podem ser uma fonte de transmissão prolongada para adultos. Mais pesquisas são necessárias para determinar a viabilidade e infectividade do SARS-CoV-2 nas fezes, de forma a controlar a disseminação do vírus.

Pouco se sabe sobre os aspectos perinatais da COVID-19. Até o momento, não há evidência de transmissão vertical intrauterina em bebês nascidos de mães infectadas por SARS-CoV-2 durante a gestação,^(12)^ mas essa possibilidade não pode ser descartada. A maioria dos neonatos sob risco de desenvolvimento de COVID-19 estudados até hoje apresentou dificuldade respiratória discreta, baixa incidência de intolerância alimentar e desfechos favoráveis.^(13)^ Além disso, o SARS-CoV-2 não parece ser excretado no leite materno. Em estudo com amostras de leite materno coletadas de seis mães, todas testaram negativo para SARS-CoV-2. Entretanto, o pequeno tamanho amostral nesses estudos impede declarações conclusivas.^(9,14)^

## MECANISMO DE INFECÇÃO POR SARS-COV-2 EM CRIANÇAS

Muitas doenças infecciosas manifestam-se de forma distinta em crianças e adolescentes e em adultos. O motivo por que a COVID-19 é menos grave em crianças permanece desconhecido. Também não se sabe se o intestino inflamado, como na colite, representa uma porta de entrada ideal para que o vírus penetre em células humanas, ou se a terapia imunossupressora tem relação com maior risco de infecção por SARS-CoV-2 em crianças e adolescentes.^(15)^ Há poucos dados a respeito.

O ponto-chave da transmissão em humanos é a capacidade do vírus de se ligar às células. O SARS-CoV-2 infecta primariamente as células epiteliais respiratórias e se dissemina principalmente desse sistema para outros órgãos. O receptor da enzima conversora de angiotensina 2 (ECA2) é essencial para células infectadas por COVID-19 e tem alta expressão em células pulmonares e em enterócitos do íleo e do cólon. O vírus utiliza a ECA2 para invadir as células. Zhu et al., relataram maior atividade de ECA2 em crianças e adolescentes com idade entre 4 e 13 anos e queda progressiva até os níveis adultos após essa idade.^(15,16)^ Os autores postularam que crianças e adolescentes podem ser menos suscetíveis à infecção por SARS-CoV-2 devido a diferenças relativas à atividade da ECA2 e à imunidade. Foi sugerido também que o SARS-CoV-2 pode se disseminar pela via fecal, devido à expressão da ECA2 em células epiteliais pulmonares e sua presença em concentrações elevadas no íleo ou no cólon.^(5,17,18)^

A síndrome da tempestade de citocinas é tida como um dos principais mecanismos patogenéticos envolvidos na falência múltipla de órgãos associada à infecção por SARS-CoV-2.^(18)^ De fato, altos níveis de expressão de citocinas e quimiocinas, como a interleucina-6 (IL-6), foram relatados em pacientes com COVID-19 e positivamente correlacionados com a gravidade da doença. Entretanto, as células-alvo, os mecanismos patogenéticos e a via de transmissão ainda não foram completamente elucidados, e muitas questões permanecem em aberto.

## CARACTERÍSTICAS CLÍNICAS DA COVID-19

A COVID-19 afeta principalmente a população adulta e causa uma série de doenças respiratórias. Os sintomas mais comuns da infecção inicial por SARS-CoV-2 são febre, tosse, fadiga, mialgia e falta de ar, sendo que 10% a 20% dos pacientes progridem para síndrome respiratória aguda grave (SARS).^(19,20)^ A idade avançada (acima de 60 anos) e a presença de comorbidades (doença cardíaca, hipertensão arterial, derrame prévio, *diabetes mellitus* e doença pulmonar ou renal crônica) são fatores de risco comuns para desfechos desfavoráveis.^(14,21)^ Os sinais e sintomas da COVID-19 em crianças e adolescentes vão de quadros assintomáticos à insuficiência respiratória aguda e são menos comuns e menos graves do que em adultos.^(4)^ Febre, tosse, falta de ar, mialgia e fadiga são os principais sintomas descritos em populações pediátricas, com manifestações graves da doença em 2% dos casos.^(4,14)^ Curiosamente, segundo os *Centers for Disease Control and Prevention* (CDC), os sintomas são semelhantes em crianças chinesas e americanas.^(21)^ Os fatores de risco para doença grave em crianças são doença pulmonar crônica, doenças cardíacas, neuromusculares ou genéticas e a imunossupressão.^(22)^ Entretanto, os lactentes parecem ser mais vulneráveis à infecção por SARS-CoV-2, e a incidência de doença grave é maior nesse grupo do que em crianças mais velhas. De fato, a prevalência de doença grave ou crítica relatada em crianças menores de 1 ano, e de 1 a 5, 6 a 10, 11 a 15 e 16 a 17 anos é de 10,6%, 7,3%, 4,2%, 4,1% e 3,0%, respectivamente.^(4)^

O choque hiperinflamatório com características semelhantes à doença de Kawasaki atípica (síndrome do choque associada à doença de Kawasaki ou síndrome do choque tóxico) foi descrito em alguns casos na Inglaterra e nos Estados Unidos. A maioria dos casos tinha apresentação clínica semelhante, com febre persistente (38°C a 40°C), *rash* cutâneo variável, conjuntivite, edema periférico, dor generalizada nas extremidades distais e sintomas gastrintestinais importantes. A evolução para choque refratário e comprometimento cardíaco é comum em afecções respiratórias específicas raras.^(23,24)^

## MANIFESTAÇÕES GASTRINTESTINAIS DA COVID-19

Sinais e sintomas gastrintestinais podem afetar 3% a 79% das crianças, adolescentes e adultos com COVID-19 e são mais comuns em casos graves.^(6,24)^ As manifestações incluem diarreia (2% a 50%), anorexia (40% a 50%), vômitos (4% a 67%), náusea (1% a 30%), dor abdominal (2% a 6%) e sangramento gastrintestinal (4% a 14%). O vômito é mais frequentemente relatado em populações pediátricas, enquanto a diarreia é o sintoma mais comum em crianças e adultos como um todo.^(25)^ Na ausência de sintomas respiratórios, a diarreia pode ser o primeiro sintoma antes do diagnóstico em 22% dos casos. É descrita a ocorrência de diarreia com fezes amarela e aquosa com frequência de três a nove vezes dejeções ao dia. O exame coprológico, em geral, revela baixa contagem de leucócitos e ausência de sangue, indicando infecção viral.^(25)^

A diarreia causada pelo SARS-CoV-2 não parece lesar o epitélio do cólon. Da mesma forma, infiltrados inflamatórios linfocitários podem eventualmente ser encontrados no esôfago, estômago, cólon e fígado de pacientes adultos com COVID-19.^(5)^ Esse achado sugere que os sintomas gastrintestinais decorrem mais da resposta imune do que de lesões orgânicas. Partindo do pressuposto de que o SARS-CoV-2 possa, de fato, disseminar-se pela via fecal, Xiao et al., enfatizaram o papel do exame de amostras de fezes por reação em cadeia da polimerase da transcrição reversa em tempo real (rRT-PCR) na prevenção da transmissão fecal-oral.^(5)^ Os pediatras e gastrenterologistas também devem permanecer vigilantes quanto a outros vírus que possam mimetizar a gastrenterite, como adenovírus, os vírus das hepatites virais, dengue e eventos gastrintestinais adversos associados à terapia imunossupressora e ao uso de agentes biológicos.

## DOENÇA INFLAMATÓRIA INTESTINAL E DOENÇA HEPÁTICA

A DII inclui doença de Crohn e colite ulcerativa. Em crianças, a DII costuma ser mais extensa e grave do que em adultos. Assim, os especialistas em DII foram desafiados a abordar aspectos específicos da infecção por SARS-CoV-2 em pacientes pediátricos com DII, principalmente aqueles em terapia imunossupressora ou biológica. Há poucos dados referentes a pacientes com DII infectados por SARS-CoV-2. As diretrizes e os consensos nacionais e internacionais sugerem que o risco de infecção por SARS-CoV-2 não é maior em pacientes com DII do que na população em geral. Em crianças e adolescentes com DII tratadas com drogas imunossupressoras que apresentem sintomas digestivos e COVID-19, devem-se primeiramente investigar as causas e excluir as infecções entéricas mencionadas. Em seguida, patógenos gastrintestinais, como *Clostridioides difficile*, devem ser excluídos. Finalmente, a presença de inflamação ativa deve ser confirmada, com base nos valores de calprotectina fecal e proteína C-reativa, ou achados de imagem em cortes transversais. O tratamento da DII deve ser realizado de acordo com a atividade e a gravidade da doença e inclui terapias imunossupressoras e biológicas. Os corticosteroides podem ser usados para tratar episódios de recorrência, desde que em doses baixas e por períodos curtos de tempo, com redução progressiva da dose o mais cedo possível.^(26,27)^ A orientação provisória de um grupo da cidade do Porto, da ESPGHAN, sugere que, em crianças e adolescentes com DII, a doença em si não é um fator de risco para infecção por SARS-CoV-2, ou para doença mais grave em relação à população em geral.^(27)^ O controle da inflamação intestinal e a manutenção das terapias são fundamentais para prevenir os desfechos adversos da DII.

Não se sabe se crianças e adolescentes com DII ou submetidos a transplante hepático e em tratamento com agentes imunossupressores ou biológicos sejam mais suscetíveis à infecção por SARS-CoV-2, e, até agora, nenhuma das duas sociedades fez qualquer recomendação.^(26,27)^ Até o momento, não há relatos de doença grave (insuficiência respiratória aguda e/ou síndrome da falência múltipla de órgãos) ou óbito entre pacientes pediátricos com DII ou transplantados hepáticos imunossuprimidos. Esses achados são derivados da experiência inicial e podem vir a mudar conforme mais dados se acumularem. As recomendações gerais e específicas e as diretrizes de tratamento para crianças com DII estão listadas nas tabelas 1 e 2, respectivamente.^(26-29)^

**Tabela 1:** Recomendações para crianças e adolescentes com doença inflamatória intestinal durante a pandemia de COVID-19

**Gerais**

Lave as mãos com frequência
Mantenha o distanciamento social (distância mínima de 1m)
Cubra a boca e o nariz ao tossir ou espirrar
Use máscara
Se tiver febre, tosse ou dificuldade de respirar, busque atenção médica rapidamente
Consultas presenciais devem ser substituídas por telemedicina sempre que possível
As equipes médicas devem monitorar os pacientes com doença ativa ou episódios de reagudização por telefone
O exercício físico é essencial e pode ser acessado em casa, pela internet
O apoio psicológico/psiquiátrico pode ser necessário

**Específicas**

Até o momento, a DII não parece ser um fator de risco para infecção por SARS-CoV-2 em crianças e adolescentes
A adesão à terapia medicamentosa deve ser reforçada
As vacinações devem estar em dia, principalmente a anual contra influenza
A doença ativa deve ser tratada de acordo com as diretrizes padrão de cuidado da DII, uma vez que os riscos se sobrepõem ao risco de complicações relacionadas à COVID-19

**Centros de infusão durante a pandemia de COVID-19**

Em crianças estáveis, a substituição do infliximabe pelo adalimumabe só deve ser realizada se a infusão intravenosa não for possível
A terapia combinada com agentes biológicos e imunomoduladores pode ser mais arriscada do que a monoterapia
Endoscopias e procedimentos cirúrgicos eletivos devem ser adiados durante a epidemia
A colonoscopia deve ser substituída pela dosagem de calprotectina fecal

Fonte: adaptado de Turner D, Huang Y, Martín-de-Carpi J, Aloi M, Focht G, Kang B, Zhou Y, Sanchez C, Kappelman MD, Uhlig HH, Pujol-Muncunill G, Ledder O, Lionetti P, Dias JA, Ruemmele FM, Russell RK; Paediatric IBD Porto group of ESPGHAN. Corona Virus Disease 2019 and Paediatric Inflammatory Bowel Diseases: Global Experience and Provisional Guidance (March 2020) from the Paediatric IBD Porto Group of European Society of Paediatric Gastroenterology, Hepatology, and Nutrition. J Pediatr Gastroenterol Nutr. 2020;70(6):727-33;^(27)^ Queiroz NS, Barros LL, Azevedo MF, Oba J, Sobrado CW, Carlos AS, et al. Management of inflammatory bowel disease patients in the COVID-19 pandemic era: a Brazilian tertiary referral center guidance. Clinics (Sao Paulo). 2020;75:e1909.^(29)^

DII: doença inflamatória intestinal; SARS-CoV-2: coronavírus da síndrome respiratória aguda grave 2.

**Tabela 2:** Considerações específicas relativas ao tratamento da doença inflamatória intestinal em pacientes pediátricos durante a pandemia de COVID-19

**Derivados do ácido 5-aminossalicílico (sulfassalazina)**

Não há evidência de risco aumentado de infecção por COVID-19
Não devem ser suspensos

**Corticosteroides**

A segurança na vigência de infecção por COVID-19 não está clara
Acredita-se que os corticosteróides sistêmicos não tragam benefícios clínicos
Os corticosteróides podem ser usados em baixa doses e por períodos curtos de tempo, para tratamento de episódios de recorrência. A dose deve ser reduzida gradualmente, assim que possível

**Imunomoduladores (tiopurinas e metotrexato)**

Não há evidência de risco aumentado de infecção por COVID-19
Os imunomoduladores foram prescritos nas doses e intervalos padrão para quase todas as crianças
SARS-CoV-2 positivos e negativos (sintomáticos): a interrupção da terapia imunossupressora é recomendada durante a doença febril aguda, devendo ser reintroduzida após a resolução da febre e restabelecimento da condição de saúde da criança^(27)^
Reiniciar o tratamento 2 semanas após a remissão dos sinais e sintomas
SARS-CoV-2 positivo (assintomáticos): as decisões terapêuticas devem ser individualizadas^(26)^

**Terapia à base de anti-TNF**

Somente infliximabe e adalimumabe foram aprovados
Não há evidência de risco aumentado de infecção por COVID-19
A dose e os intervalos de infusão devem ser mantidos
SARS-CoV-2 positivo e assintomático: as terapias biológicas devem ser postergadas por 2 semanas para monitoramento dos sintomas de COVID-19 e reiniciadas após a remissão dos sinais e sintomas^(26)^
A substituição do infliximabe pelo adalimumabe deve ser desencorajada em pacientes estáveis

Fonte: adaptado de Rubin DT, Feuerstein JD, Wang AY, Cohen RD. AGA clinical practice update on management of inflammatory bowel disease during the COVID-19 Pandemic: expert commentary. Gastroenterology. 2020 Apr 10:S0016-5085(20)30482-0. doi: 10.1053/j.gastro.2020.04.012. [Epub ahead of print];^(26)^ Turner D, Huang Y, Martín-de-Carpi J, Aloi M, Focht G, Kang B, Zhou Y, Sanchez C, Kappelman MD, Uhlig HH, Pujol-Muncunill G, Ledder O, Lionetti P, Dias JA, Ruemmele FM, Russell RK; Paediatric IBD Porto group of ESPGHAN. Corona Virus Disease 2019 and Paediatric Inflammatory Bowel Diseases: Global Experience and Provisional Guidance (March 2020) from the Paediatric IBD Porto Group of European Society of Paediatric Gastroenterology, Hepatology, and Nutrition. J Pediatr Gastroenterol Nutr. 2020;70(6):727-33;^(27)^ Queiroz NS, Barros LL, Azevedo MF, Oba J, Sobrado CW, Carlos AS, et al. Management of inflammatory bowel disease patients in the COVID-19 pandemic era: a Brazilian tertiary referral center guidance. Clinics (São Paulo). 2020;75:e1909.^(29)^

SARS-CoV-2: coronavírus da síndrome respiratória aguda grave 2; TNF: fator de necrose tumoral.

## ENVOLVIMENTO E RECOMENDAÇÕES NUTRICIONAIS

Curiosamente, em crianças e adolescentes com formas graves de COVID-19, o início agudo da tempestade de citocinas pró-inflamatórias pode levar à desnutrição e à sarcopenia em um curto período de tempo. A sarcopenia foi associada à mortalidade ou à morbidade pós-operatória em diversos procedimentos cirúrgicos.^(30,31)^

A diarreia e o vômito foram descritos em 9% e 7% dos pacientes, respectivamente, e acredita-se que tenham papel importante no desenvolvimento de subnutrição, um fator de risco em pacientes críticos.^(30)^ Entretanto, a importância da transmissão fecal permanece desconhecida, assim como a da relação entre a expressão de ECA2 no sistema digestivo e a invasão das células epiteliais pelo vírus.^(14,32,33)^

Em pacientes adultos, a perda de peso decorrente da falta de apetite é muito comum (78,5%) e pode agravar a condição nutricional nas formas graves da doença. A anosmia pode comprometer o paladar e interferir na ingestão de suplementos nutricionais ou alimentos.^(33,34)^

O choque e a condição clínica grave associados à doença causada pelo novo coronavírus não devem ser vistos como contraindicações para a nutrição enteral trófica. Em crianças e adolescentes com comprometimento respiratório importante e necessidade de suporte ventilatório, pode ocorrer a deglutição excessiva de ar com exacerbação da distensão gástrica.^(15,30)^ Essa condição pode predispor ao refluxo gastresofágico e à pneumonia aspirativa associada à ventilação mecânica. A infecção pulmonar pode causar atraso no esvaziamento gástrico e à hipomotilidade intestinal, causando constipação. Tais condições clínicas impedem a instituição imediata de terapia nutricional. Além disso, a mensuração da tolerância alimentar, com base no volume gástrico residual ou em outros indicadores, é altamente controversa, assim como a influência do tipo de paciente (cirúrgico ou não). A associação de outros parâmetros clínicos (distensão, vômito e peristaltismo normal) é mais confiável. Entretanto, a alimentação gástrica é preferível para otimizar a atividade digestiva, sendo a via pós-pilórica (preferencialmente a jejunal) recomendada em caso de falha. O uso de agentes procinéticos é recomendado para estímulo da motilidade antes desse passo. Professionais da saúde devem ter cuidado na realização procedimentos que possam gerar aerossóis (sondas pós-pilóricas carregam risco mais alto). Dietas poliméricas (com ou sem lactose) e terapias nutricionais oligoméricas especiais (principalmente a administração pós-pilórica) são usadas com frequência. A associação entre nutrição enteral contínua e controle da diarreia foi frisada em alguns estudos.^(15,30,35-37)^

As dietas enriquecidas com lipídios podem satisfazer as necessidades calóricas e evitar a depleção de ácidos graxos essenciais ou a produção excessiva de dióxido de carbono. Dietas pobres em fibras podem ser mais bem toleradas na presença de disfunção grastrintestinal importante. Não há evidência de que a suplementação de ômega 3, arginina ou glutamina possa resultar em melhor evolução nutricional ou prognóstico em populações pediátricas.^(15,30,38,39)^

Em pacientes com COVID-19 e comprometimento respiratório grave, a superalimentação deve ser evitada, devido ao maior risco de hipercapnia. Decúbito ventral não é uma contraindicação para a nutrição enteral precoce e foi associado a um melhor prognóstico.^(14,31)^

O fornecimento de níveis mínimos de proteína de 1 a 2,5g/kg ao dia é necessário para evitar o hipercatabolismo proteico.^(14)^ As necessidades de micronutrientes podem ser maiores em pacientes com COVID-19. Entretanto, a suplementação adicional com altas doses de vitaminas (incluindo as vitaminas A, C, D e E) ou de outros oligoelementos, como o zinco e o selênio, não é recomendada. Estudos investigando as necessidades de micronutrientes em adultos com COVID-19 são controversos.^(15,39)^

A nutrição parenteral deve ser evitada, exceto em pacientes com subnutrição grave ou insuficiência intestinal. A administração gástrica facilita a instituição precoce da alimentação. A nutrição enteral precoce parece ser a melhor recomendação para garantir a evolução antropométrica adequada em pacientes pediátricos com COVID-19. O fornecimento suficiente de alimentos só pode ser determinado por meio de avaliações nutricionais sequenciais, incluindo achados clínicos e laboratoriais.^(15,39)^ O leite materno é um verdadeiro imunonutriente, e sua administração deve ser estimulada.

Até o momento, as recomendações referentes à terapia nutricional são mais baseadas em dados empíricos do que em evidências derivadas de ensaios clínicos e devem seguir os princípios básicos de cuidados nutricionais para pacientes críticos.^(14)^

Durante a pandemia de SARS-CoV-2, é fundamental que as necessidades nutricionais das crianças e adolescentes vulneráveis sejam satisfeitas para evitar a exacerbação das disparidades, em termos de saúde e progresso educacional. Mais tempo diante de telas, horário irregular de sono, dietas de pior qualidade que resultam em ganho de peso e perda de condição cardiorrespiratória são efeitos secundários do fechamento das escolas e do confinamento doméstico em crianças.^(36,40,41)^

## DADOS LABORATORIAIS RELACIONADOS À COVID-19

Alguns achados laboratoriais específicos são dignos de nota em pacientes com COVID-19. A elevação dos níveis de aminotransferase afeta 20% a 30% dos pacientes. A hipoproteinemia e o aumento do tempo de protrombina também foram relatados durante a infecção por COVID-19. Queda da contagem de monócitos e maior prevalência de antibioticoterapia foram relatados em pacientes adultos com COVID-19 e sintomas digestivos, em relação a pacientes sem sintomas digestivos. A linfopenia, um mau indicador prognóstico, parece ser menos comum em crianças.^(30,37,38)^

Concentrações elevadas de proteína C-reativa, procalcitonina, citocinas, ferritina, triglicérides e dímeros D foram descritas nos casos citados de choque hiperinflamatório, com evidência laboratorial de infecção ou inflamação grave.^(24)^

## DISCUSSÃO E AVALIAÇÃO CRÍTICA

No início da pandemia de COVID-19, os sintomas digestivos não foram priorizados. Com a evolução da pandemia e a coleta de dados acumulados em escala global, ficou evidente que os sintomas digestivos são, de fato, muito comuns.^(5,6)^

A detecção do SARS-CoV-2 em amostras de fezes e em outros espécimes clínicos trouxe à tona o risco de transmissão pela via fecal-oral. Foi sugerido também que o sistema digestivo possa ser uma via de infecção por COVID-19, principalmente entre crianças e adolescentes, embora essa hipótese ainda não tenha sido confirmada. Além disso, as amostras de fezes coletadas de crianças permanecem positivas por mais tempo do que as de adultos.^(7,8,39)^

Crianças e adolescentes com doenças gastrintestinais crônicas, como DII e doença hepática, podem ter risco maior de infecção por COVID-19 do que indivíduos saudáveis. Entretanto, os bancos de dados de vigilância, diretrizes e consensos provisórios internacionais sugerem que essas doenças não sejam um fator de risco nesses pacientes.^(27,28)^ Faltam dados acumulados para mais esclarecimentos.

Crianças e adolescentes com COVID-19, principalmente os que desenvolvem a forma grave da doença, podem sofrer rápida deterioração do estado nutricional. A instituição precoce de nutrição enteral, por meio de sondas gástricas cuidadosamente colocadas para minimizar o risco de contaminação pessoal, é fundamental. A nutrição parenteral aumenta muito o risco de internação prolongada por infecções secundárias. A sarcopenia com prognóstico desfavorável pode se desenvolver em resposta à ventilação mecânica prolongada. A terapia nutricional pode contribuir para um melhor prognóstico e recuperação, e não há evidência de maiores riscos de diarreia persistente ou manifestações gastrintestinais, desde que a dieta fornecida seja adequada.^(40-42)^

O sistema digestivo pode ser uma via potencial de transmissão da infecção pelo novo coronavírus. Crianças e adolescentes com sintomas gastrintestinais, como vômito e diarreia, devem ser consideradas suspeitas de COVID-19. Faltam estudos para determinar se a transmissão pela via fecal-oral de fato ocorre. Entretanto, a possibilidade de que pacientes infectados excretem o vírus nas fezes não pode ser descartada.

Doenças crônicas, como a DII e doença hepática, não parecem ser um fator de risco para a COVID-19. Da mesma forma, até o momento, não há indicação para suspensão da terapia imunossupressora nos pacientes afetados.

A terapia nutricional é essencial para evitar desnutrição e sarcopenia em casos graves. Novos estudos são necessários para investigação de manifestações gastrintestinais, achados de alterações laboratoriais e de imagem em grandes populações de crianças e adolescentes afetados pela COVID-19.
